# Elastic Properties of Polychloroprene Rubbers in Tension and Compression during Ageing

**DOI:** 10.3390/polym12102354

**Published:** 2020-10-14

**Authors:** Rami Bouaziz, Laurianne Truffault, Rouslan Borisov, Cristian Ovalle, Lucien Laiarinandrasana, Guillaume Miquelard-Garnier, Bruno Fayolle

**Affiliations:** 1 Centre des Matériaux, Mines ParisTech, PSL University, CNRS UMR 7633 BP 87, F-91003 Evry, France; rami.bouaziz@mines-paristech.fr (R.B.); cristian.ovalle_rodas@mines-paristech.fr (C.O.); lucien.laiarinandrasana@mines-paristech.fr (L.L.); 2 PIMM, Arts et Metiers Institute of Technology, CNRS, Cnam, HESAM University, 151 boulevard de l’Hôpital, 75013 Paris, France; lauriane.truffault@ensam.eu; 3 EDF, Nuclear New Build Engineering & Projects, Design and Technology Branch, La Grande Halle, 19 rue Pierre Bourdeix, 69007 Lyon, France; rouslan.borisov@edf.fr

**Keywords:** polychloroprene, ageing, mechanical properties, rubber elasticity theory, crosslinking

## Abstract

Being able to predict the lifetime of elastomers is fundamental for many industrial applications. The evolution of both tensile and compression behavior of unfilled and filled neoprene rubbers was studied over time for different ageing conditions (70 °C, 80 °C and 90 °C). While Young’s modulus increased with ageing, the bulk modulus remained almost constant, leading to a slight decrease in the Poisson’s ratio with ageing, especially for the filled rubbers. This evolution of Poisson’s ratio with ageing is often neglected in the literature where a constant value of 0.5 is almost always assumed. Moreover, the elongation at break decreased, all these phenomena having a similar activation energy (~80 kJ/mol) assuming an Arrhenius or pseudo-Arrhenius behavior. Using simple scaling arguments from rubber elasticity theory, it is possible to relate quantitatively Young’s modulus and elongation at break for all ageing conditions, while an empirical relation can correlate Young’s modulus and hardness shore A. This suggests the crosslink density evolution during ageing is the main factor that drives the mechanical properties. It is then possible to predict the lifetime of elastomers usually based on an elongation at break criterion with a simple hardness shore measurement.

## 1. Introduction

Rubbers (also called elastomers) are loosely crosslinked networks of amorphous polymers having a very low glass transition temperature [1]. Due to their macromolecular structure, these materials are relatively soft (Young’s modulus on the order of 1–10 MPa) but display a large deformability (up to about 10 times their original length), mostly reversible, due to an elasticity that is entropic in nature [2]. Amongst this class of materials, polydiene-based elastomers such as polyisoprene or polychloroprene (neoprene) have remarkable mechanical properties (especially a large elongation at break) along with a good stability towards chemicals [3]. However, their use is often limited by the fact that they harden and become brittle over the long term. These changes in mechanical properties are most often caused by an oxidation phenomenon [4]. Indeed, the oxidation process leads to the production of free alkyl/peroxyde radicals along the chain which can then react with the double bonds by addition [5]. These inter-macromolecular addition reactions then contribute to an increase in the crosslinking density [6].

The classical way to predict the lifetime of elastomers is to consider that the time to reach an end-life criterion in terms of elongation at break (such as 50% of initial elongation at break) follows a pseudo-Arrhenius behavior. Based on accelerated ageing time test, an extrapolation at room temperature can then be done to predict lifetime. However, this kind of extrapolation is highly questionable since there are many experimental results showing that end life does not always follow such an empirical law [7].

To avoid such extrapolation, a possible way is to develop an approach in two steps. The first step is to build a kinetic model taking into account all the chemical mechanisms involved during ageing: according to this model, it is then possible to simulate the chemical state and the chain scission/crosslinking events whatever the exposure conditions in terms of time or temperature of exposure (see for instance for the polychloroprene [8]). The second step is to relate the chemical state and the chain scission/crosslinking events to the relevant property such as elongation at break or hardness. This kind of structure–property relationship has to be valid whatever the accelerated ageing conditions to be used in real conditions. 

The prediction of the lifetime of elastomer parts first requires reliable constitutive models to be established in 3D, i.e., taking the hydrostatic pressure into account. The basic Mooney–Rivlin deformation energy *W* commonly used to assess the lifetime of engineering elastomeric structures is expressed as Equation (1) [9]:

(1) $$W=C_{10}\left(I_{1}-3\right)+C_{01}\left(I_{2}-3\right)+0.5K{\left(J-1\right)}^{2}$$

where *C*_10_, *C*_01_ and *K* are the material parameters, *K* being in particular the bulk modulus of the elastomer; *I*_1_ and *I*_2_ the first and second invariants of the deformation gradient tensor; *J* the Jacobian which can be written as the volume ratio *V*/*V*_0_, with *J* = 1 for perfectly incompressible materials.

Deriving the uniaxial Cauchy stress σ from Equation (1) leads to:

(2) $$\sigma =\frac{1}{J}\left(2C_{10}+\frac{2C_{01}}{\lambda }\right)\left(\lambda^{2}-\frac{1}{\lambda }\right)+K\left(J-1\right)$$

with *λ* the elongation, defined as *λ* = l/l_0_ where l_0_ is the initial sample length.

The relationship between hyperelastic (Mooney–Rivlin) material parameters (*C*_10_, *C*_01_), bulk modulus (*K*) and elastic properties at small strain (Young’s modulus *E* and Poisson’s ratio *ν*) can then be summarized as follows:

(3) $$E=3\left(2C_{10}+2C_{01}\right)$$

(4) $$K=\frac{E}{3\left(1-2v\right)}$$

In this context, relating the evolution of both hyperelastic and elastic material properties to the progress of the crosslinking process is one of the objectives of this study, since it is required for lifetime prediction of the elastomers.

In the case of unfilled elastomers, the value of the Poisson’s ratio is often considered to be equal to 0.5, corresponding to an incompressible state of the material, i.e., a deformation that occurs at constant volume [10,11]. However, elastomers are actually “ever so slightly” compressible and it is noteworthy that measuring the Poisson’s ratio is not straightforward. In the case of filled elastomers, the value of the Poisson’s ratio has been typically measured between 0.46 and 0.49 [12,13,14]. By using Equation (4) and considering a typical elastomeric Young’s modulus of 10 MPa, such variation of the Poisson’s ratio leads to an increase of the bulk modulus *K* from 42 MPa to 167 MPa. These values are in contradiction with the supposed incompressibility assumption letting the bulk modulus *K* tends to infinity, and a small uncertainty in the Poisson’s ratio measured value can easily lead to an uncertainty of as much as an order of magnitude for *K*. 

To the best of our knowledge, there are no results available allowing us to characterize such a pair of hyperelastic and elastic parameters during an oxidative crosslinking, which is then required to perform finite elements simulations of industrial parts. In the literature, the changes in mechanical behavior during the crosslinking process induced by oxidation are characterized mainly by tensile tests to assess *E* [15] or toughness in mode I [16]. However, although different loading conditions are investigated through these experiments, none of them gives access simultaneously to two parameters as described above. Therefore, a relevant mechanical characterization is required for a proper assessment of *E* and *ν* (or *K*), which can monitor not only the changes in terms of Young’s modulus during the crosslinking process due to ageing, but also the possible changes in terms of compressibility modulus or Poisson’s ratio.

In this study, the objective is to characterize the mechanical behavior of filled and unfilled crosslinked polychloroprene through tensile and oedometric compression tests to assess both the change of *E* and *ν* (or *K*) over time at different oxidative conditions. The choice of several oxidative conditions has been driven to check possible relationships between these values independently of oxidation rate, i.e., temperature of exposure. A specific attention is paid to promote spatial homogeneous oxidation for the samples considered here in order to assess only intrinsic material relationships.

Finally, the evolution of the elastic properties *E* and *K* during ageing is compared to elongation at break or hardness which are classically used to follow oxidative ageing of elastomers. The second objective of the study is then to propose correlations between all these mechanical properties, which shall be valid not only during the whole oxidation process but also for both unfilled and filled elastomers. As a result, a critical value for a specific property (easy to measure, such as hardness shore A) could be used as a proxy for critical values of properties that are much harder to assess (such as elongation at break), for lifetime prediction purposes.

## 2. Materials and Methods

Thin rectangular plates (thickness = 2 mm) of unfilled neoprene rubber and of neoprene filled with 40 phr (~30 wt %) of carbon black were provided by Nuvia (Villeurbanne, France). To study the impact of the thermal oxidative ageing on the mechanical properties, the plates were aged at three different temperatures (70 °C, 80 °C and 90 °C) during specific ageing times. It was verified with microindentation tests that ageing was homogeneously distributed throughout the samples (see Appendix A). All samples were thoroughly characterized after each ageing time. Specifically, differential scanning calorimetry (DSC) was used to follow the consumption of antioxidants/stabilizers under oxidative ageing. Mechanical tests, namely tensile, hardness, and oedometric tests, were developed to follow the evolution of the mechanical properties, namely Young’s modulus, elongation at break and bulk modulus, with the ageing time. All mechanical tests were carried out at ambient temperature.

### 2.1. DSC/OIT

The antioxidants/stabilizers consumption was analyzed through the determination of the oxidation induction time (OIT) measured with a Q20 DSC from TA Instruments (New Castle, DE, USA). Polychloroprene samples with a mass ranging between 7.0 and 8.0 mg were placed in aluminum standard pans (TA Instruments) without a lid. Reference consisted of an empty aluminum pan, also without a lid. After equilibrating the temperature at 50 °C and an isothermal of five minutes, the samples were heated to 180 °C at 10 °C/min under nitrogen flow at 50 mL/min. Temperature was then maintained at 180 °C for five minutes before substituting nitrogen for oxygen for 250 min. OIT corresponds to the intersection of the tangent of the baseline at the time of atmosphere change with the tangent at the beginning of the oxidation peak. 

### 2.2. Tensile Tests 

The tensile tests were conducted on H3 dog-bone specimens (4 mm wide, 2 mm thick and 20 mm long obtained with a cutting-die) with a 5966 Instron tensile machine (Instron, Élancourt, France). Two samples were tested per ageing condition. Forces were monitored using a load cell of 10 kN. The specimens were stretched up to failure with a crosshead speed set to 20 mm/min. Due to the ability of the samples to undergo very large strains, pneumatic clamping jaws were used to avoid slippage.

Young’s modulus was determined by fitting the stress–elongation curves in the *λ* = [1; 1.45] region with a Mooney–Rivlin incompressible model in uniaxial tension, i.e., following Equations (2) (adapted for engineering stress) and (3).

### 2.3. Hardness Tests (Shore A) 

The hardness of the material was measured using a shore A durometer (model LX-A-Y from RS PRO, Corby, UK). The measured values indicate the resistance to indentation of the tested material on a scale between 0 (depth of indentation equal to the sample thickness) and 100 (no indentation).

### 2.4. Oedometric Compression Test

The oedometric compression tests were conducted on discs (diameter = 25 mm and thickness = 2 mm) obtained from the rubber plates with a cutting die. A stack of three discs was inserted in a lab-made apparatus consisting in a steel die with a compression applied by means of a rigid stamp. A small gap (less than 0.5 mm) between the samples and the steel die was initially set to allow the specimens to be positioned inside the die. The compression load was applied with a tensile/compression Instron machine (model 5982, Instron France) with a load cell of 100 kN. The specimens were compressed to about 95 kN with a displacement speed set to 1 mm/min.

## 3. Results

### 3.1. Chemical Stability: OIT Changes

**Figure 1:**
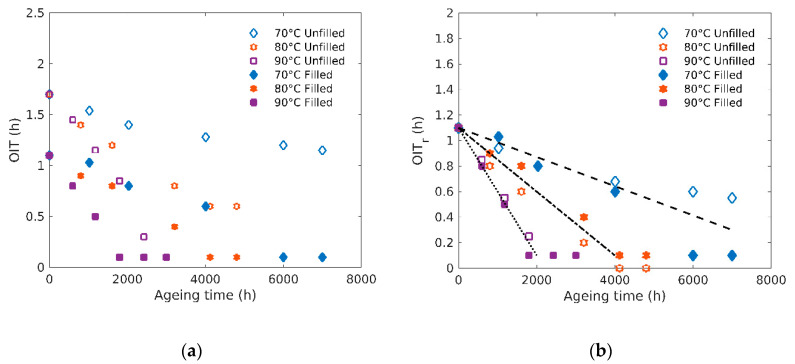
(**a**) Oxidation induction time (OIT) and (**b**) relative OIT (OIT_r_) evolution as a function of ageing time.

Figure 1a reports the changes of OIT as a function of ageing time for filled and unfilled polymers at the three temperatures of exposure. The initial OIT gap between unfilled and filled samples can be attribute d to different processing conditions leading to a higher antioxidants loss in the case of the filled samples, such as a supplementary blending step at high temperature, using an extruder or an internal mixer. 

As expected, OIT decreases with ageing time, indicating a consumption of the antioxidants/stabilizers during the samples exposure. OIT reaches a plateau value close to 0 for the filled rubbers indicating an almost total consumption of the antioxidants. This plateau is reached at 2000–3000 h for samples aged at 90 °C and after about 6000 h for the filled samples aged at 70 °C, while it was not obtained for the unfilled samples aged at the same temperature during the total time of the experiment (8000 h).

In order to take into account the fact that unfilled sample shows a higher initial OIT value, we plot here in Figure 1b “relative OIT” (OIT_r_) which is defined equal to the actual OIT for the filled samples, and for the unfilled values as OIT − 0.6 (which is the OIT difference at t = 0 h between unfilled (1.7) and filled (1.1) samples). Thus, Figure 1b shows the relative OIT for the three temperatures of exposure and allows to compare both elastomers in terms of OIT decrease. It clearly appears that the relative OIT decrease rate (i.e., the slope of the “linear” region shown as dotted lines in Figure 1b) follows the same trend for both elastomers whatever the exposure temperature. As a result, filled and unfilled elastomers show the same ageing kinetic and differ only by their initial state. 

A classical way to quantify the relative OIT change during ageing as a function of temperature is to consider the slope *x* of the linear region follows a pseudo-Arrhenius behavior with temperature [7], i.e., x=x0eEaRT with *E_a_* the activation energy (J/mol), R the gas constant = 8.314 J/mol/K and *T* the temperature (in kelvin). *E_a_* is then considered as a characteristic of the process and its activation by temperature. Here, the temperature of exposure activates the OIT drop rate with an activation energy close to 78 kJ/mol considering such behavior.

### 3.2. Tensile Tests

**Figure 2:**
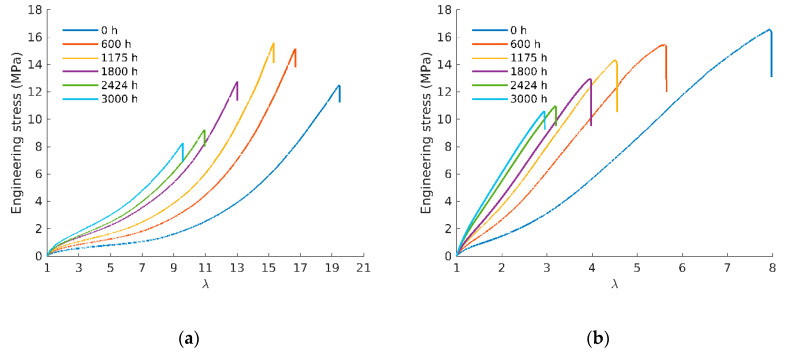
(**a**) Tensile curves to failure of samples aged at 90 °C for unfilled and (**b**) filled rubbers.

Figure 2 displays representative tensile curves for unfilled (a) and filled (b) samples aged at 90 °C and tested at different ageing times. The plots show the engineering stress (the applied load divided by the unstretched cross-sectional area) as a function of the elongation *λ*.

In Figure 2a, it can be observed that the unfilled rubber undergoes a very large strain, leading to a pronounced hardening before failure. For polychloroprene rubbers, it has been reported that this hardening is due to strain-induced crystallization (SIC) [17]. As proposed in [18], SIC-related stress hardening during tensile test appears amplified by the presence of fillers (see Figure 2b). This, along with the filler properties, leads to the samples initially stiffer but with a smaller elongation at break than the unfilled rubbers. Both SIC onset associated to the stress–elongation curve upturn and elongation at break decrease monotonously with ageing. Furthermore, the upturn phenomenon is only clearly visible for the nonaged sample and disappears for the aged samples. However, all tensile curves show a similar trend with ageing: stiffening and elongation at break decrease, both trends being attributed to extra crosslinking induced by ageing. 

**Figure 3:**
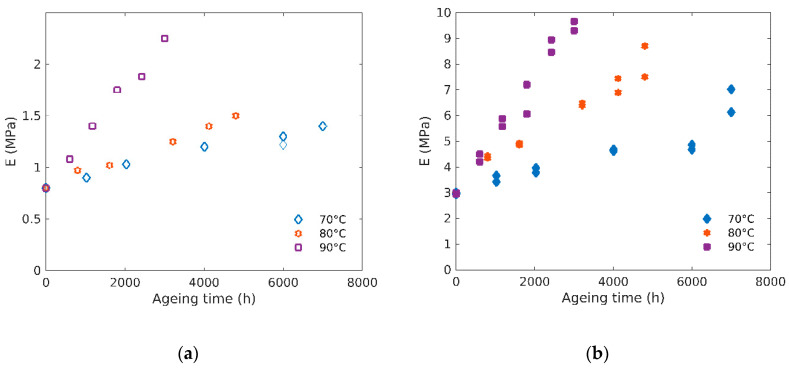
(**a**) Evolution of Young modulus (*E)* with ageing time for unfilled and (**b**) filled rubbers at 70 °C, 80 °C and 90 °C.

Moduli changes can be extracted from the stress–elongation curves for both rubbers at all exposure conditions. Figure 3 shows the evolution of the Young’s modulus (*E*) with ageing time for unfilled (a) and filled (b) rubbers. This confirms that *E* continuously increases with ageing and that this increase is more pronounced at higher temperatures of exposure for a given ageing time. Similar trends have been obtained on a carbon black filled butadiene in [19]. Since modulus is driven by the crosslink density, it is thus possible to follow chemical modification through this quantity. Activation energy for the modulus (calculated using the same procedure as the one described for the OIT assuming here an Arrhenius behavior) increase rate is close to 90 kJ/mol for the unfilled and 84 kJ/mol for the filled rubber. 

**Figure 4:**
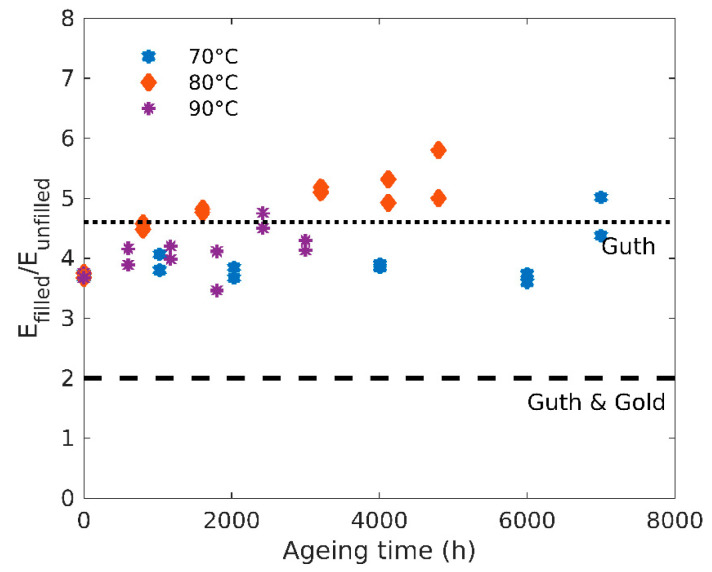
Ratio of the filled and unfilled moduli as a function of ageing time for three ageing temperatures.

In order to study the fillers’ effect on the modulus evolution, the ratio of the filled and unfilled moduli is plotted as a function of ageing time at the three temperatures in Figure 4. It appears this ratio is almost constant for all temperatures and ageing time, with a value of about 4.5. It is worth noting that this value is higher than the Guth and Gold equation [20,21] given by Equation (5):

(5) $$\frac{E_{filled}}{E_{unfilled}}=1+2.5\varphi +14.1\varphi^{2},$$

where *φ* is the effective volume fraction of filler. 

Assuming a density of 2 g/cm^3^ for carbon black and 1.2 g/cm^3^ for the elastomer [22], the volume fraction of fillers here is about 0.2, which yields a value of 2 for the modulus ratio using Equation (5).

A modified equation by Guth [20] takes into account the shape factor *f* of the carbon black (see Equation (6)):

(6) $$\frac{E_{filled}}{E_{unfilled}}=1+0.67f\varphi +1.62f^{2}\varphi^{2}$$

With a shape factor *f* = 6.5 as determined by Mullins and Tobin [23], Equation (6) gives a value of 4.6 for modulus ratio, consistent with the experimental data, as can be seen in Figure 4. 

**Figure 5:**
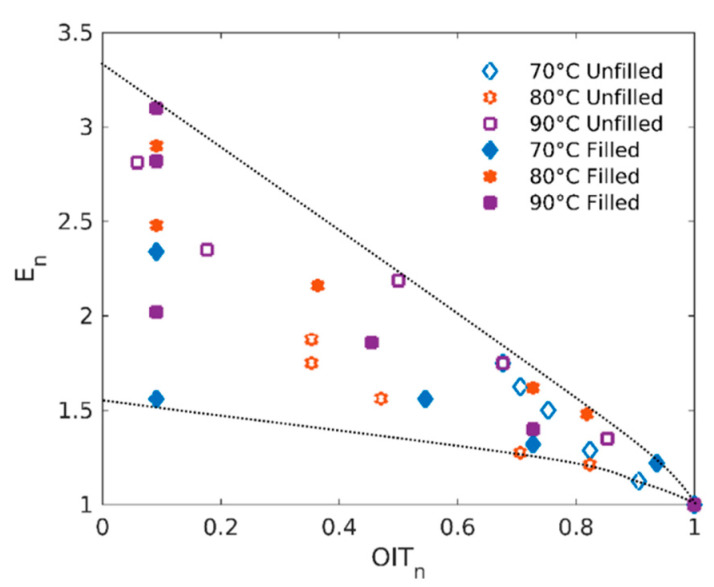
Normalized modulus E_n_ as a function of normalized oxidation induction time OIT_n_.

Finally, if we consider modulus changes as a tracer of the chemical degradation, a correlation between chemical stability (i.e., OIT) and the evolution of normalized Young’s modulus (defined as *E*(*t*)/*E*(*t* = 0)) shall be evidenced. Figure 5 presents normalized modulus as a function of normalized OIT (OIT_n_ = OIT(*t*)/OIT(*t* = 0)) for both filled and unfilled rubbers at the three ageing temperatures. All the data display a similar trend and fall within the same envelope (see dotted lines in Figure 5): starting from high normalized OIT values (unaged rubbers), normalized Young’s modulus increases as normalized OIT decreases until a limit value of OIT which corresponds to a total consumption of stabilizers (OIT_n_ reaching values close 0). 

**Figure 6:**
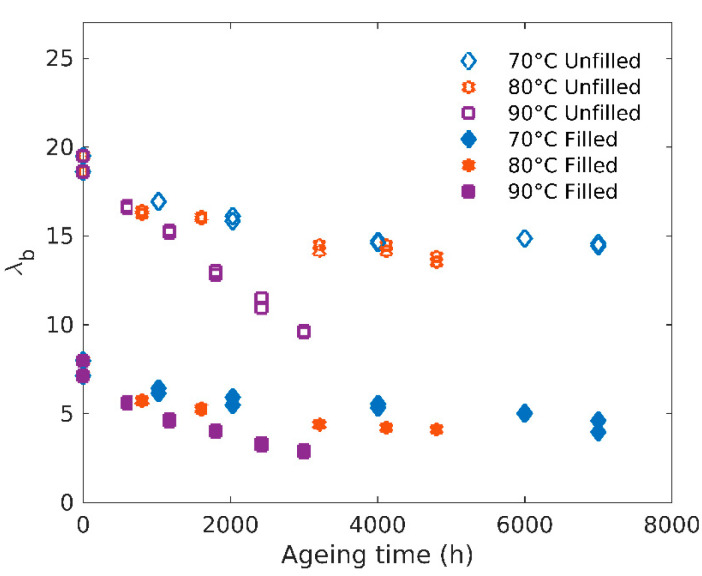
Evolution of the elongation at break with ageing time.

From Figure 2, it is also possible to extract the elongation at break evolution, which is a relevant parameter often used as a criterion to define end-life duration in industrial applications of such materials [24]. As expected, Figure 6 shows the elongation at break decreases with ageing, again in a much more pronounced way at higher temperatures. 

This embrittlement process is necessarily associated with the crosslink density increase already witnessed through modulus changes. From a kinetic point of view, elongation at break decrease rate shows an activation energy close to 86 kJ/mol for the unfilled and 74 kJ/mol for the filled rubber, consistent with the values obtained for the moduli.

### 3.3. Hardness Tests (Shore A) 

**Figure 7:**
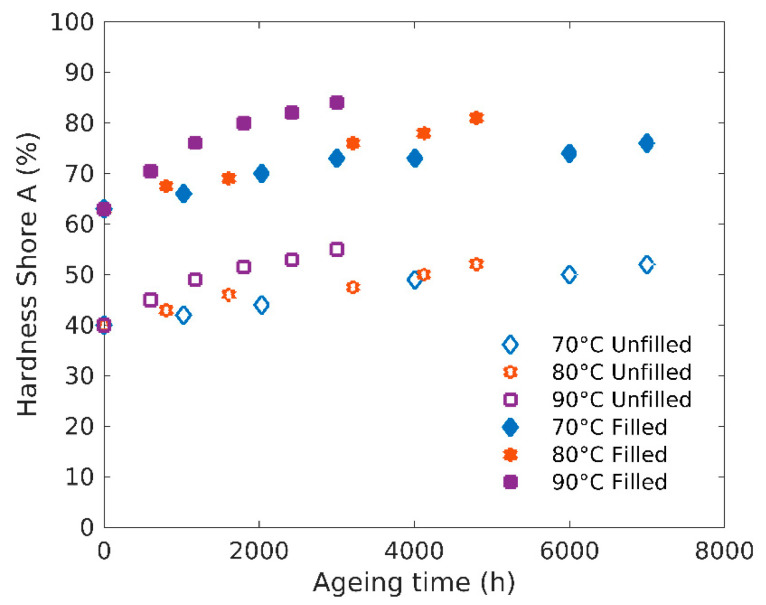
Hardness Shore A evolution with ageing time.

Although values obtained from hardness tests are a priori too qualitative to identify the elastic parameters, they are easy to obtain and as such often used in the industry. Figure 7 shows the evolution of the materials hardness with ageing time. It appears that the hardness of the rubbers increases with ageing similarly to the Young’s modulus. 

### 3.4. Bulk Modulus 

The small-strain elastic mechanical behavior of isotropic materials is fully characterized by a couple of parameters, for example Young’s modulus (*E*) and bulk modulus (*K*). If the evolution of the Young’s modulus with ageing is often reported in the literature [15], no data concerning such evolution of the bulk modulus of elastomers are available according to our knowledge.

**Figure 8:**
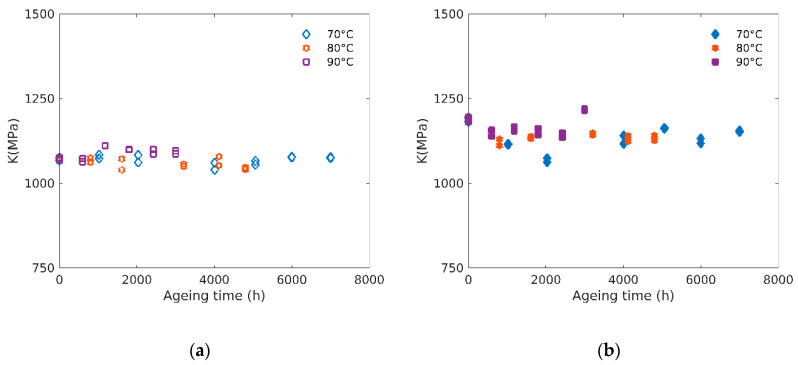
(**a**) Evolution of the bulk modulus (*K*) with ageing time for unfilled and (**b**) filled rubbers at 70 °C, 80 °C and 90 °C.

Figure 8 shows such evolution for unfilled (see Figure 8a) and filled (b) rubbers. The values obtained are in the GPa range, similar to previous reports for elastomers [25]. Contrary to what has been observed for Young’s modulus (see Figure 3), the effect of ageing on the bulk modulus is small both in terms of time and temperature dependency (i.e., less than 5% variations). It can also be noticed that the bulk moduli of filled rubbers are slightly higher than the ones of unfilled rubbers (from about 1100 MPa to 1200 MPa).

Let us recall Young’s modulus can be related to the crosslink density, whereas bulk modulus is essentially controlled by van der Waals forces [26,27]. Chemical degradation yielding a crosslinking process does not modify significantly the van der Waals’ energy.

## 4. Discussion

### 4.1. Structure-Properties Relationships

The Young’s modulus of rubbers can be related to the crosslink density following Equation (7):

(7) E=3NρRT.

with N the crosslink density in mol/kg and *ρ* the elastomer density (kg/m^3^) [28]. As a consequence, the crosslink density modifications which can be deduced from *E* measurements may be linked to OIT, elongation at break and hardness changes. Although FTIR measurements only slightly evidence oxidation products in the timeframe of the ageing experiments (see infrared spectra in Appendix B), it is well known that oxidation leads to crosslinking in the case of polychloroprene rubbers during long-term exposure in air, due to addition reactions between alkyl/peroxyl radicals and double bonds [6,8]. This is consistent with the fact that OIT decreases when *E* increases. 

To confirm this, the activation energies estimated in the previous section for all these properties are listed and can be compared in Table 1: it appears that all activation energies are close to 80 kJ/mol, suggesting that all these properties changes have the same chemical origins and are driven by the same mechanisms. In [4], similar activation energy characterizing the degradation was found (~90 kJ/mol), the values for the unfilled neoprene also being slightly higher than those of the filled material. Finally, note that the hardness shore A activation energy value is a bit smaller for the unfilled samples but that is probably due to experimental uncertainty as the variations of hardness over ageing time are very small for these samples. 

Let us also examine direct correlation between *E* and hardness. Many relationships between *E* and hardness shore A (*S_A_*) are proposed in the literature [29,30]. In [30], a finite element simulation study leads to a linear relation between the logarithm of the elastic modulus and the hardness values (see Equation (8)):

(8) $$\log_{10}E=c_{1}\;S_{A}-c_{2}$$

with *c*_1_ and *c*_2_ empirical constants determined from the best fit as *c*_1_ = 0.0235 and *c*_2_ = 0.6403 for 20 < *S_A_* < 80 [30].

**Figure 9:**
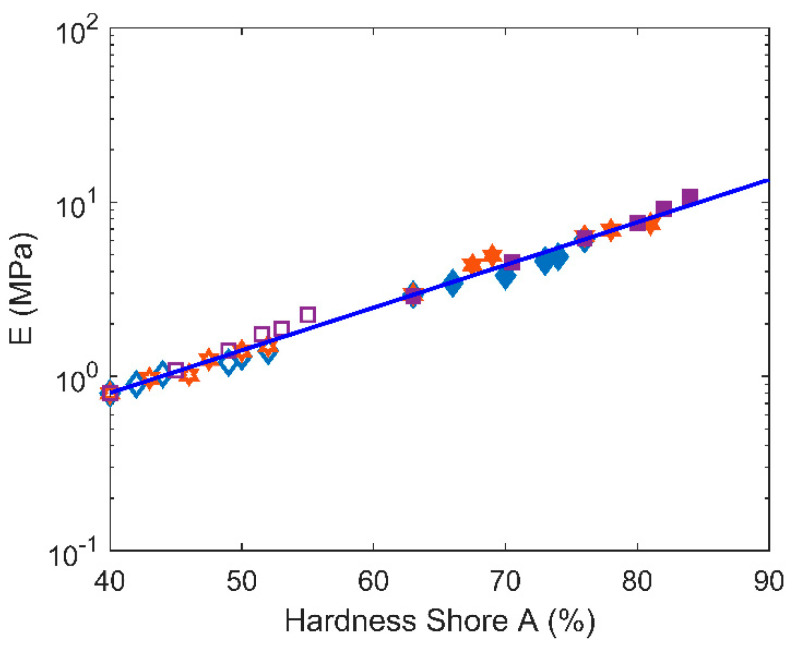
Young’s modulus (E) versus hardness shore A for all samples (filled, unfilled) and temperatures.

In Figure 9, *E* is plotted as a function of *S_A_*. According to the previous approach, our experimental results lead to *c*_1_ = 0.0245 and *c*_2_ = 1.075 for both filled and unfilled samples, independently of ageing, in the 40 < *S_A_* < 85 range, which is in good agreement with the values reported previously [30].

Finally, we would like now to study possible correlations between *λ_b_* and *E*. As a first approximation, the elongation at break can be related to the chain dimensions [1]: assuming an initial Gaussian conformation $R_{0}=\sqrt{n}l$ with *R_0_* the chain end-to-end distance, *n* the average number of repeating units in the chain and *l* the monomer typical size, and fracture occurring when the chain extension reaches a maximal value $R_{\max}=nl$, we can write Equation (9):

(9) $$\lambda_{b}\propto \frac{R_{\max}}{R_{0}}=\sqrt{n}$$

**Figure 10:**
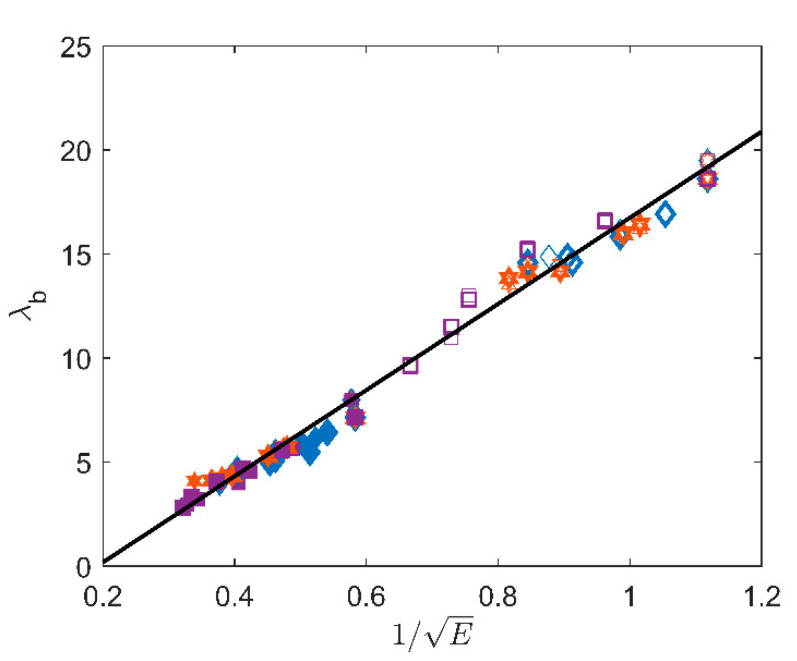
$\lambda_{b}$ versus $1/\sqrt{E}$ for all samples (filled, unfilled) and temperatures.

Similarly, the Young’s modulus of rubbers can be related to the molar mass between crosslinks, according to Equation (7) rewritten under the form $E=3\frac{\rho RT}{M_{c}}$ with *M_c_* the molar mass between crosslinks (in kg/mol), which is proportional to *n*. Hence, $\lambda_{b}$ should scale as $1/\sqrt{E}$ which is indeed observed in Figure 10. Interestingly, this simple scaling remains valid whatever the ageing conditions and for both filled and unfilled materials. 

The linear fit gives the following value with E in MPa: $\lambda_{b}=20.7/\sqrt{E}-4$.

Assuming again a density of 1.2 g/cm^3^, and with a molar mass *M_0_* of 88 g/mol for the polychloroprene monomer, the calculated prefactor is $\sqrt{\frac{3\rho RT}{M_{0}}}\approx 10$ MPa^−1/2^. This is in very good agreement with the value obtained from the fit. Note the calculated value is obtained by assuming the typical molar mass between crosslinks is the molar mass of the whole chain, which explains the slightly underestimated value obtained. 

To conclude this part, correlations between *E*, *λ_b_* and hardness shore A show a direct relation according to simple physical relations from rubber elasticity theory. Interestingly, these correlations are valid for the filled and unfilled rubbers during ageing. From a practical purpose, the choice of a critical value for one of these three properties can be simply translated in terms of crosslink density changes during ageing.

### 4.2. Poisson’s Ratio

All mechanical properties determined here have to allow the description of the behavior law which can be, for example, later used in a finite element code: *E* and *v* or the shear modulus *G* and *K* for instance. The final objective would be to perform simulations to predict strain and stress in an elastomer part under loading and after ageing. 

**Figure 11:**
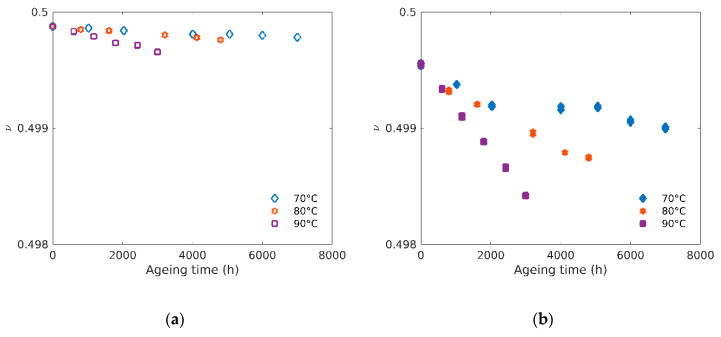
(**a**) Poisson’s ratio versus ageing time for unfilled and (**b**) filled rubbers at 70 °C, 80 °C and 90 °C.

Figure 11 shows the evolution of the Poisson’s ratio (*v*) with ageing time of both unfilled (a) and filled (b) rubbers aged at three different temperatures. Values of *v* were obtained using the following relationship that links *E* and *K* (see Equation (4), rewritten as follows): v=0.5 (1−E/3K).

It can be noted again that the often used approximation for rubbers *v* = 0.5 is an upper limit (see Figure 11) obtained when *K* tends to infinity. For this reason, this limit value cannot be used in finite elements simulations.

We can observe a small linear decrease of *v* from 0.4996 to 0.4986 at 90 °C and 0.4988 at 80 °C in the case of filled rubbers. However, in the case of unfilled rubbers (and of filled rubbers at 70 °C), the decrease of *v* may be considered as negligible, with a value of 0.4995 ± 0.0004. To the best of our knowledge, this is one of the first measurements of the evolution of the Poisson’s ratio with ageing for elastomers. 

## 5. Conclusions

In this article, we described the evolution of Young’s modulus, elongation at break and hardness shore A as a function of ageing conditions. In this study, we also developed an experimental protocol allowing to estimate the Poisson’s ratio evolution for the same sample. If the Poisson’s ratio is only marginally affected by ageing for the unfilled rubbers, a slight decrease is witnessed in the case of the filled rubbers. This point, previously unseen in the literature, has to be taken into account for 3D element finite simulations of aged rubber industrial parts. 

It is shown that Young’s modulus and elongation at break can be quantitatively linked to each other using simple scaling arguments coming from the rubber elasticity theory, whatever the samples (filled or unfilled) and ageing time and temperature. This strongly suggests that the mechanical properties evolution is mainly driven by the extra crosslinking of the neoprene matrix, induced by ageing.

Since Young’s modulus and hardness are also related quantitatively to each other, it can then be possible to predict the lifetime of elastomers based on an elongation at break criterion with a simple hardness shore measurement. This result may have numerous applications for industries where predicting such lifetime is of fundamental importance. This precise measurement of the Poisson’s ratio could also open the way to more quantitative mechanical models. 

## Figures and Tables

**Table 1:** Activation energy for all parameters understudied.

*E_a_* (kJ/mol)	E	OIT	λ_b_	Hardness
Unfilled	90	78	86	53
Filled	84	78	74	72

**Figure A1:**
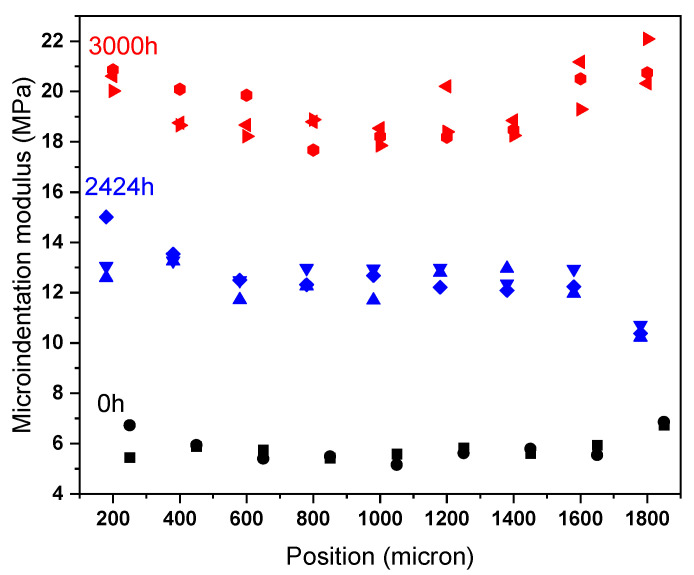
Evolution/profile of modulus through the thickness measured by microindentation of a 2 mm-thick sample aged at 90 °C for different durations.

**Figure A2:**
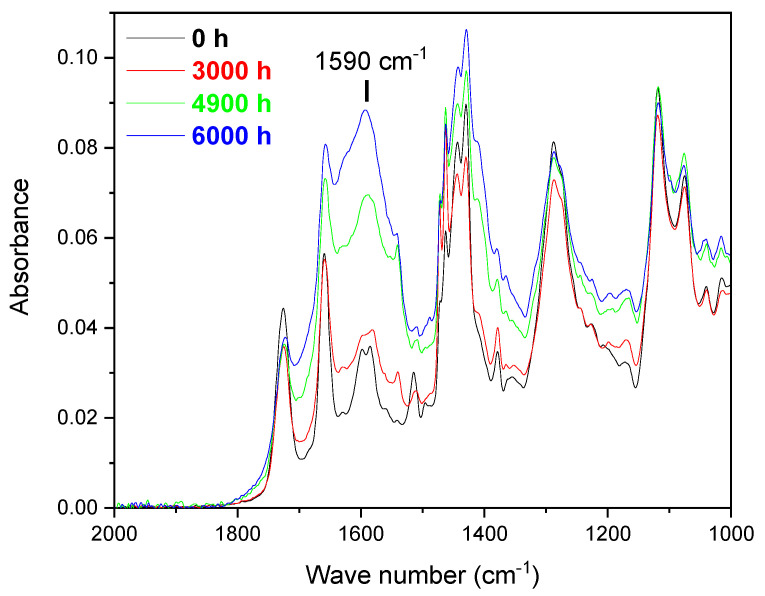
FTIR spectra of neoprene aged at 90 °C for different durations.

## Appendix A

Small pieces of neoprenes (about 10 mm long, 5 mm large and 2 mm thick) were cut and embedded in epoxy resin. The epoxy embedded neoprenes were then polished in order to obtain flat areas. Local moduli were measured each 200 μm along the neoprenes thickness with a microhardness tester from CSM + Instruments equipped with a conospherical indenter. Hertz’s model was used to determinate the modulus from the loading region of the strength–indentation depth curves.

The modulus profiles along the thickness measured by microindentation for three filled neoprenes aged at 90 °C during different times are presented in Figure A1. For the most aged sample (3000 h), the highest modulus increase between the center of the sample and its edge was around 17%. This value was slightly higher (33%) for the unaged sample. Our results demonstrate the absence of oxidation profile within the samples thickness. Indeed, these increases are both very small compared to the increases reported by Wise et al. [31] for neoprene aged at 100 °C (>100% increase) and by Le Gac et al. who reported a modulus approaching 1 GPa in the 100 μm superficial zone for neoprene aged at 120 °C [32].

Furthermore, it is interesting to note that the ratio between the average moduli measured by microindentation and the average moduli measured by traction is similar for the different exposition conditions (~1.5–2).

## Appendix B

Unfilled neoprene plates were analyzed by Fourier transform infrared spectroscopy (FTIR) in attenuated total reflectance (ATR) mode using a Frontier FTIR spectrometer from Perkin Elmer. Spectra were obtained in the 4000–650 cm^−1^ region with a resolution of 4 cm^−1^ and 16 scans.

The spectra of neoprene aged at 90 °C for different times are presented in Figure A2. The spectral range between 2000 and 1000 cm^−1^ is of particular interest when studying polychloroprene degradation due to the presence of bands attributed to oxidation products [4,5,32]. It should be noted that unfilled neoprene has been aged up to 6000 h for this analysis, while in the rest of the study the longest ageing time was 3000 h. The intensity of the band centered at 1590 cm^−1^ clearly increases with ageing, but the change becomes only significant after 4900 h. This band can be attributed to carboxylate species. Their presence results from the conversion of acid chlorides and carboxylic acids during ageing in the presence of a small amount of metal oxides such as ZnO, classically used as an activator for the sulfur vulcanization of rubbers [33].
